# Prescription glucocorticoid medication and iridocyclitis are associated with an increased risk of senile cataract occurrence: a Mendelian randomization study

**DOI:** 10.18632/aging.205963

**Published:** 2024-06-26

**Authors:** Rui Wen, Yu-Jia Xi, Ran Zhang, Si-Jia Hou, Jin-Yu Shi, Jin-Yi Chen, He-Yi Zhang, Jun Qiao, Yi-Qian Feng, Sheng-Xiao Zhang

**Affiliations:** 1 Department of Rheumatology, The Second Hospital of Shanxi Medical University, Taiyuan, Shanxi, China; 2 Shanxi Provincial Key Laboratory of Rheumatism Immune Microecology, Taiyuan, Shanxi, China; 3 Key Laboratory of Cellular Physiology at Shanxi Medical University, Ministry of Education, Taiyuan, Shanxi, China; 4 Department of Neurology, First Hospital of Shanxi Medical University, Taiyuan, Shanxi, China; 5 Department of Breast Surgery, Fifth Hospital of Shanxi Medical University, Taiyuan, Shanxi, China

**Keywords:** Mendelian randomization, prescription glucocorticoid medication use, iridocyclitis, senile cataracts, single nucleotide polymorphisms

## Abstract

Iridocyclitis and the use of glucocorticoid medication have been widely studied as susceptibility factors for cataracts. However, the causal relationship between them remains unclear. This study aimed to investigate the causal relationship between the development of iridocyclitis and the genetic liability of glucocorticoid medication use on the risk of senile cataracts occurrence by performing Two-sample Mendelian randomization (MR) analyses. Instrumental variables (IVs) significantly associated with exposure factors (P < 5 × 10^-8^) were identified using published genome-wide association data from the FinnGen database and UK Biobank. Reliability analyses were conducted using five approaches, including inverse-variance weighted (IVW), MR-Egger regression, simple median, weighted median, and weighted mode. A sensitivity analysis using the leave-one-out method was also performed. Genetic susceptibility to glucocorticoid use was associated with an increased risk of developing senile cataracts (OR, 1.10; 95% CI, 1.02-1.17; P < 0.05). Moreover, iridocyclitis was significantly associated with a higher risk of developing senile cataracts (OR, 1.03; 95% CI, 1.01-1.05; P < 0.05). Nonetheless, some heterogeneity in the IVs was observed, but the MR results remained consistent after penalizing for outliers. The estimates were consistent in multivariate analyses by adjusting for body mass index (BMI) and diabetes mellitus type 2 (T2DM). This study provides new insights into the prevention and management of senile cataracts by highlighting the increased risk associated with iridocyclitis and the use of glucocorticoids.

## INTRODUCTION

Senile cataracts are the most common diseases that cause temporary or permanent blindness [1]. It affects about 21% of the global population [2]. With economic growth and an aging population, the number of blind people is increasing [2, 3]. Although it is a treatable disease, the socioeconomic effects of cataract surgery and the psychological burden on patients after surgery are enormous [3]. Since there is no consensus manual for preventing senile cataracts, preventing disease progression effectively remains challenging. Several risk factors have been reported to accelerate the development of senile cataracts. Among them, the induction of uveitis and the use of cortisol hormones have attracted the attention of many researchers [1, 3–5].

Iridocyclitis, the most common type of uveitis, produces inflammation involving the iris and ciliary body in the anterior part of the uvea [6, 7]. The incidence and prevalence of elderly patients are the highest [7]. Cataracts and elevated eye pressure often complicate it [8]. Cortisol, the most widely used of glucocorticoids, is the most preferred option for treating iridocyclitis [9–11]. Classical studies have shown that glucocorticoids induce cataracts while eliminating inflammation [11, 12]. However, some contradictory points still emerged in the recent cohort study. Active inflammation was more likely to lead to cataracts than corticosteroid use [13]. Long-term follow-up revealed that cortisol doses were used in greater amounts in eyes that did not develop cataracts [13]. Conflicts may arise due to bias caused by unadjustable confounding factors in the observed data. Therefore, the role of glucocorticoid use and iridocyclitis inflammation in cataract induction remains to be investigated. Although prospective randomized clinical trials (RCT) are the criterion for the inference of cause and effect [14], trials are complicated to conduct due to the fact that assessing the impact of drug use often coincides with the inflammatory effects of the disease.

Mendelian randomization (MR) is a method for evaluating the causality of risk factors on the disease by using genetic variants as instrumental variables (IVs) to substitute risk factors [15]. Because genetic variants are randomly assigned at conception, potential confounding factors are avoided. Concurrently, MR minimizes the risk of reverse causality on account of the assignment of single nucleotide polymorphic alleles prior to the onset of meiosis [16, 17]. MR plays an essential role in the inference of causality when RCT is impractical to achieve [18].

Consequently, in this study, we designed a two-sample MR analysis. Summary data on self-reported use of prescribed glucocorticoids from the UK Biobank (UKB) [19], and iridocyclitis and senile cataract data from the FinnGen database were used to conduct univariable MR (UVMR) analyses and multivariate MR (MVMR) analyses to investigate the potential causal relationship between genetic liability for glucocorticoids and iridocyclitis, respectively, for senile cataract.

## RESULTS

### Instrument variables

In total, 19 SNPs were included as instrument variables (IVs) for iridocyclitis and 21 independent SNPs as IVs for glucocorticoid medication use (Supplementary Tables 1, 2). There was no weak instrumental variation because the f-statistic values of the SNP selected in the MR analysis were more than 100 (Supplementary Tables 1, 2). We found no reverse causality SNPs using steiger filtering analysis, indicating that the causal relationships obtained based on IVs are reliable. MR-PRESSO detected an SNP (rs6941966) with a potential pluripotent outlier in the IVs used for iridocyclitis and deleted it. The MVMR analyses used a total of 729 IVs. We performed fitting regularized regression models and identified 695 genetic variants as valid instruments. Only valid genetic variants were used to estimate causal effects using standard IVW.

### UVMR analysis

**Figure 1:**
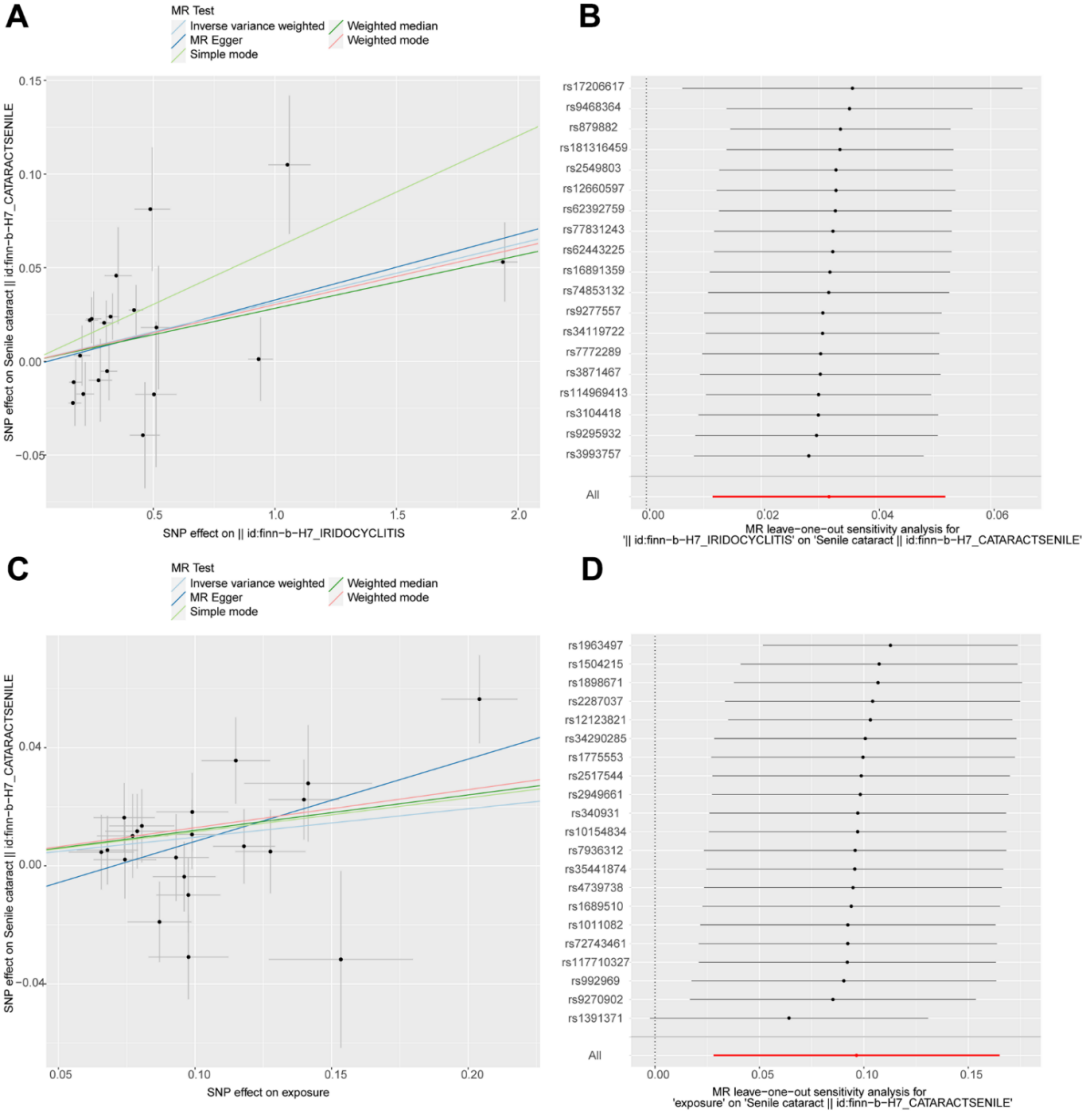
**Scatterplot and leave-one-out analysis of iridocyclitis and glucocorticoid on the risk of developing senile cataracts.** IVs indicating age-related cataract were plotted against 2 mutually independent samples showing glucocorticoid use and iridocyclitis for MR analysis and sensitivity analysis. (**A**) Scatter plot illustrating the association between iridocyclitis and senile cataracts; (**B**) show leave-one-out analysis plot of iridocylitis on senile cataracts; (**C**) scatter plot illustrating the association between glucocorticoid use and senile cataracts; (**D**) show leave-one-out analysis plot of glucocorticoid use on senile cataracts; each point in the scatter plot (**A**, **C**) represents the IV, the lines on the IVs represent confidence intervals in the vertical and horizontal directions, and the horizontal and vertical coordinates represent the effect of the IVs on exposure and outcome, respectively. The different colored lines in the figure indicate the fitting effect of different methods on MR results. Leave-One-Out sensitivity analysis (**B**, **D**). Each black point represents the estimate of iridocyclitis and glucocorticoid medication use level on senile cataracts after the corresponding single nucleotide polymorphism (SNP) was excluded. MR, Mendelian randomization; IVs, instrumental variables.

The results of the univariate analysis showed that the development of iridocyclitis (IVW OR, 1.03; 95% CI, 1.01-1.05; P < 0.05) and the use of glucocorticoids (IVW OR, 1.10; 95% CI, 1.02-1.17; P < 0.05) both increase the risk of developing senile cataracts. All MR analyses were generally consistent (Figure 1).

Afterward, we performed a series of sensitivity and pleiotropy analyses. The MR-Egger intercept test showed no significant pleiotropy (Supplementary Figures 1, 2). We also did not find outliers in the Leave-One-Out sensitivity analysis (Figure 1). Meanwhile, Cochran’s Q test indicated that IVs associated with using prescribed glucocorticoid analogs were not heterogeneous in both IVW and MR_egger models. It is unfortunate that for the MR analysis of iridocyclitis and cataract, Cochran’s Q test found heterogeneity in IVs (Table 1).

**Table 1:** Results of MR postoperative heterogeneity and polymorphism analysis.

	**Heterogeneity**			**Pleiotropy**	
	**Method**	**Q_df**	**Q_pval**	**Egger_intercept**	**pval**
Glucocorticoiden	MR Egger	19	0.12350969	—	—
	IVW	20	0.06905899	-0.01963879	0.1134135
Iridocyclitis	MR Egger	17	0.01550132	—	—
	IVW	18	0.02138715	-0.002578342	0.744869

Q df, Q statistic degrees of freedom; Q P-value, Q statistic P-value; pval, P-value of global test; IVW, inverse-variance weighted.

Although the weighted median model is the superior model for inferring causality, in this case, suggesting that the results are still statistically significant, we still choose to penalize the outliers. Since the p-value in the intercept test was insignificant and the p-value in Cochran’s Q test was greater than 0.05, we inferred the existence of balanced pleiotropy [20]. Balanced pleiotropy means that the pleiotropic effects of genetic instruments are balanced around the overall effect [20]. To ensure the reliability of the results, we used the Mendelian randomization R package to penalize abnormal IVs causing heterogeneity and perform a more robust regression analysis in IVW and MR-Egger analysis. After penalizing the abnormal values, all MR analyses showed a significant causal relationship between iridocyclitis and senile cataract (Table 2).

**Table 2:** MR analysis of iridocyclitis and senile cataracts in the presence and removal of abnormal values.

**MR analysis of heterogeneity**			
**Method**	**Estimate Std**	**95% CI**	**P-value**
IVW	0.031	0.011—0.051	0.003
MR-Egger	0.035	0.004—0.066	0.026
(intercept)	-0.003	-0.018—0.013	0.741
Weighted median	0.028	0.009—0.048	0.005
**MR analysis of heterogeneity after penalization**			
**Method**	**Estimate Std**	**95% CI**	**P-value**
Penalized robust IVW	0.031	0.016—0.046	0.000
Penalized robust MR-Egger	0.034	0.014—0.054	0.001
(intercept)	-0.002	-0.016—0.012	0.779
Penalized weighted median	0.028	0.008—0.048	0.005

MR, Mendelian randomization; IVW, inverse-variance weighted; Estimate Std, Estimate standard deviation; 95% CI, 95% Confidence interval.

### MVMR analysis

**Figure 2:**
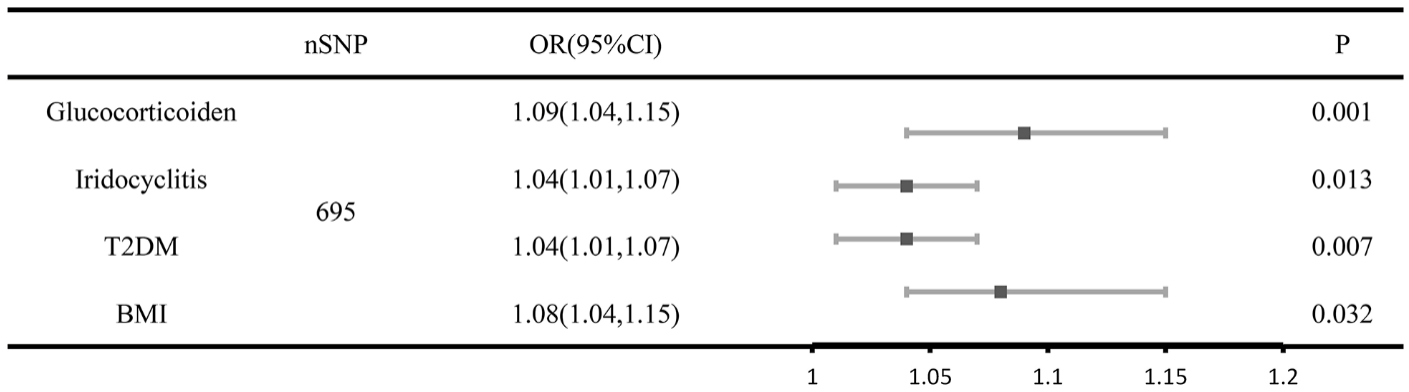
**Forest plot of MVMR analysis of senile cataracts after adjusting for risk factors taking into account iridocyclitis and prescription glucocorticoid drug use.** The causal effect of iridocyclitis, prescription glucocorticoid medications, BMI and T2D on the risk factors of senile cataract based on the multivariate analyses. Error bars represent 95% confidence intervals CI, confidence interval; OR, odds ratio; SNPs, single-nucleotide polymorphisms.

In multivariate analyses, iridocyclitis attenuated the genetic liability for the development of senile cataracts (Figure 2) whereas prescription glucocorticoid medications enhanced the genetic liability for the development of senile cataracts (Figure 2). We first adjusted for common risk factors associated with senile cataract for UVMR to identify that body mass index (BMI) induces senile cataract development and that diabetes mellitus type 2 (T2DM) mellitus similarly increases the risk of senile cataract development (Supplementary Figure 3). The Egger intercept indicates no horizontal polytropy in the MVMR analysis (P > 0.05).

## DISCUSSION

In the present study, we investigated the potential causal relationship between prescription glucocorticoid medications and iridocyclitis in senile cataract. We concluded, in agreement with most previous studies, that the use of prescription glucocorticoids and the development of iridocyclitis are both risk factors for the development of senile cataract disease. When iridocyclitis was considered alongside glucocorticoid medication use in MVMR analyses, controlling for confounders, the genetic liability for inflammation and medication use remained associated, with a diminished genetic liability for inflammation and an enhanced genetic liability for medication use.

Several previous observational studies have shown that iridocyclitis predisposes to cataracts, especially for senile cataracts, where complications can be more complex [1, 13, 21–23]. Nevertheless, it is challenging to separate inflammation from confusion in the observational analysis. Lotti et al. designed a trial in which 80% of the patients were treated with glucocorticoids [24]. Inadequate sample size and recall bias weakened the reliability of the study conclusions [13]. There is evidence in our findings that iridocyclitis can be a risk factor for cataract development. The five MR analyses showed that iridocyclitis and senile cataracts were consistent in magnitude and direction and that the MR-Egger intercept was close to 0, indicating a lack of pleiotropy. The physiological mechanisms by which iridocyclitis promotes the development of senile cataracts are still unclear. Recurrent episodes of iridocyclitis may lead to a decrease in aqueous pH, which disrupts the epithelial Na^+^/K^+^ ATP enzyme pump and leads to increased lens permeability [24, 25]. It has also been reported that uveitis can lead to cataract formation by oxidation due to exposure to subpopulations of lens proteins [26]. In our experiments, we used PhenoScanner to query the mutants’ corresponding proteins, then searched for protein annotations in the Uniport database and checked 20 SNPs as tool variables. Our results revealed that the SKIV2L gene corresponding to rs114969413, the ERAP1 gene corresponding to rs2549803, the PRRC2A corresponding to rs3993757, and the HLA-DPB1 gene corresponding to rs9277557 are all closely associated with the inflammatory process, which further demonstrates that the inflammatory process of iridocyclitis is one of the important factors leading to cataract development [27–30]. This is a special contribution to explain the mechanism of cataracts caused by iridocyclitis.

As for glucocorticoids, it has been used to treat eye diseases for almost 50 years. Glucocorticoids lead to therapeutic and side effects by converting the glucocorticoid receptor in the cytoplasm to a conformationally released activating or inhibiting signal. H Nida Sen et al. researchers designed the trial 13.8% of the 914 patients who received periocular injections were treated with cataract surgery during the follow-up period [31]. Jick et al. also found that with the increased use of inhaled glucocorticoid prescription drugs, individuals over the age of 40 were more likely to develop cataracts, while for younger individuals, the rate of increased risk was not significant [32]. This is consistent with our view that the use of glucocorticoid prescription therapy drugs can increase the risk of senile cataract development. Since glucocorticoids are routinely used for the treatment of iridocyclitis, we performed MVMR analyses. Firstly, after elimination of multiple covariates, the results suggest that bias due to inflammation on the outcome is unlikely in the current study. Secondly, the genetic variants used in this study only predicted genetic susceptibility to drug use, which results reminds us that the potential risk of drug use on the development of senile cataracts should be considered in clinical use even for therapeutic doses. There are many hypotheses regarding the mechanism of action of glucocorticoids causing cataracts, including the inhibition of the Na^+^/K^+^ pump leading to the aggregation of lens proteins [33] and the occurrence of glucocorticoid-lens protein conjugates leading to subcapsular clouding [34]. Also, the risk of glucocorticoid-induced senile cataract development may be related to intraocular penetration, treatment volume, and treatment duration. One study has shown that inhaled glucocorticoid use for more than two years increases the risk of cataract development [35]. Another study found the development of cataractous lens clouding even after reducing the use of glucocorticoids. It is interesting to note that further damage to the crystal can be prevented when the drug is discontinued [36]. Clarifying the risk of glucocorticoid use for the development of senile cataracts has positive implications for clinical prevention efforts.

Our innovative research has several advantages. We used the largest sample size for GWAS data analysis, and the larger the sample size, the more accurate the results. A complementary MR approach was used to evaluate MR analysis violations of assumptions. We also set strict thresholds to maximize the reliability of the IVs. There are also some limitations in our study. There are also some limitations in our study that need to be treated with caution. Firstly, we only studied European populations, and our findings should be interpreted cautiously for other populations. Secondly, we did not classify the timing and mode of drug use and only described the genetic liability of drug use in relation to senile cataracts. Future genetic studies will use more detailed information on drug use to strengthen causal inferences.

## MATERIALS AND METHODS

### Study design

**Figure 3:**
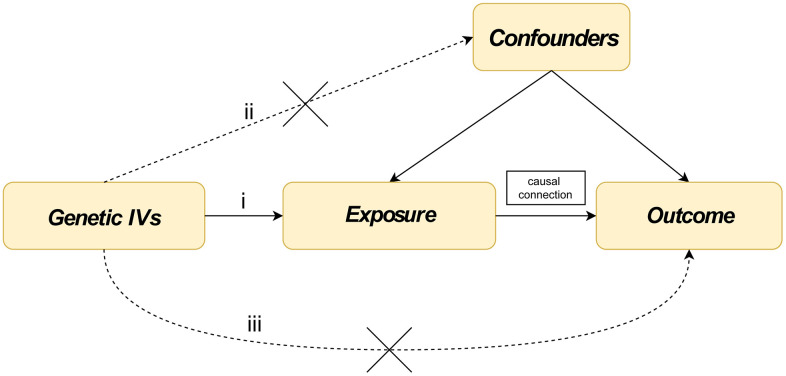
**Three assumptions about glucocorticoid medication use, senile cataract and iridocyclitis in this Mendelian randomization study.** (**i**) The instrument variables (IVs) are strongly associated with iridocyclitis and glucocorticoid medication use; (**ii**) the genetic IVs are independent of confounders; (**iii**) the genetic IVs affect senile cataract only through their effect on iridocyclitis and glucocorticoid medication use, and not through an alternative causal pathway.

The study used all publicly available summary-level data and did not require additional institutional review board ethical approval. The study design satisfies the three assumptions of MR: (i) the instrument variables (IVs) are strongly associated with the outcome; (ii) the genetic IVs are independent of confounders; (iii) the genetic IVs affect senile cataract only through their effect on exposure and not through an alternative causal pathway (Figure 3).

### Study populations

Summary data on the use of glucocorticoids were obtained from a genome-wide association research (GWAS) analysis study of self-reported drug use in the UKB [19]. A total of 502,616 people were included in this study, including about 54% of the female participants, but the number of women taking the drug increased with age. We cataloged the drugs by effective component using an anatomical therapeutic chemical classification system. Glucocorticoids included in the study consisted of Beclomethasone Dipropionate, Fluticasone Propionate, Budesonide, Mometasone, Betamethasone, Triamcinolone, and Flunisolide. We also used summary data of 3622 individuals with iridocyclitis in the FinnGen database, including 2019 female and 1603 male patients. The classification criteria for the case data of patients with iridocyclitis included in the study were derived from the International Classification of Disease-10 (ICD-10). Some of the iridocyclitis patient categories include diseases of the iris and ciliary body classified elsewhere: ankylosing spondylitis, tuberculosis, herpes zoster, tuberculosis, syphilis (secondary), herpesviral (herpes simplex) infection, and gonococcal infection. Other patients with iridocyclitis include acute and subacute iridocyclitis chronic iridocyclitis, lens-induced iridocyclitis, and other iridocyclitis. Data were adjusted for age, sex, and the top 10 genetic principal components. FinnGen data from 26758 individuals were used for the outcome data, excluding individuals with ambiguous sex, high genotypic deletions (> 5%), and excessive heterozygosity (±4 SDs). Geriatric cataract cases were defined by H25 in ICD-10), FinnGen ICD-8 37402 cases. IVs for BMI were derived from a meta-analysis of 681,275 individuals of European ancestry by Genetic Investigation of Anthropometric Traits (GIANT) [37]. Information on genetic factors related to T2DM was obtained from the Diabetes Genetics Replication and Meta-analysis (DIAGRAM) consortium [38].

### Sample independence

Overlap of exposure and outcome samples may lead to outcome bias and inflated type I error rates [39]. The analysis of glucocorticoid drug use was derived from two different cohort studies, avoiding the effect of sample overlap. Since the sample overlap between iridocyclitis and senile cataract was <10% and the strength of the IVs for iridocyclitis was considered strong (F>100), weak instrumental bias and inflation of the type I error rate were not expected [39].

### Selection of genetic IVs

**Figure 4:**
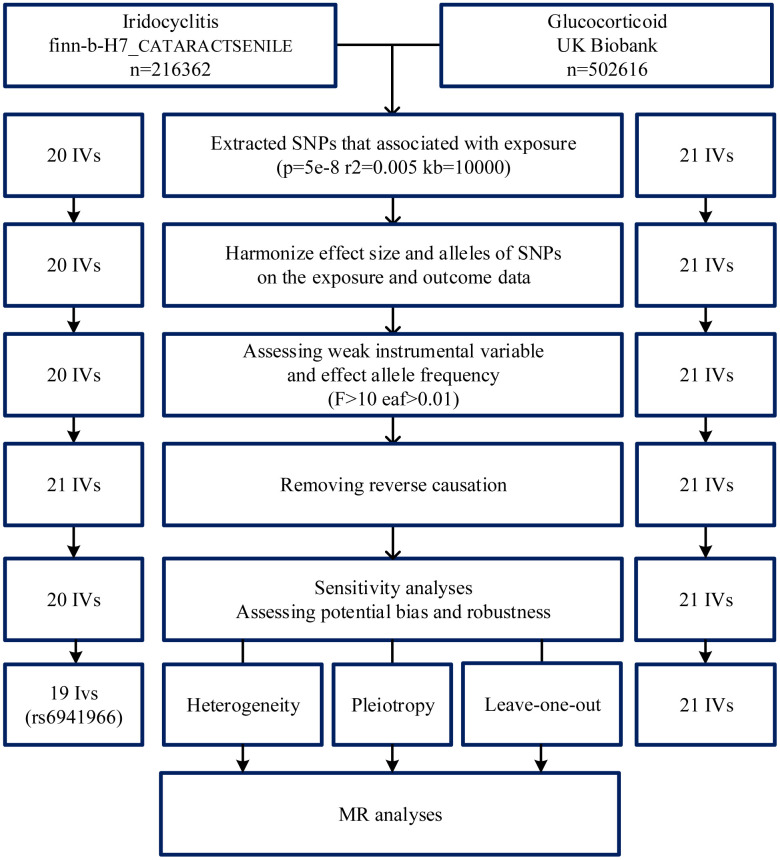
**Flowchart of instrumental variables (IVs) screening.** GWAS, genome-wide association study; MR, Mendelian randomization; SNP, single nucleotide polymorphism; IVs, instrumental variables.

To ensure the reliability of the results, the IVs were selected to satisfy the three assumptions of the Mendelian analysis [40]. First, we set a relatively strict threshold of P (P < 5 × 10^-8^). Independent IVs can be singled out using Linkage disequilibrium (LD) (r^2^ < 0.005). We used LDlink to find a proxy single nucleotide polymorphism (SNP) if the SNP was unavailable (https://ldlink.nci.nih.gov/). Secondly, we calculated the F-statistic for each SNP to exclude the effect of weak IVs on the results of F-statistics > 10. Thirdly, according to Mendel’s third hypothesis, we removed IVs that were significantly expressed in the results and deleted SNP with mutation frequencies greater than 1% after testing the allele frequencies of all SNP. To exclude outliers from the data, we used MR-pleiotropy residual sum and outlier (MR-PRESSO) prior to MR analysis (Figure 4).

### Statistical analysis

The study was analyzed in R (version 0.5.2) using TwoSampleMR and MendelianRandomization R package in the MR-Base platform. We used five reliability analysis methods: Inverse inverse-variance weighted (IVW), MR-Egger regression, simple median, weighted median, and weighted mode.

In UVMR, the IVW analysis method is valued for us. IVW uses an inverse weighting approach to calculate estimates of specific ratios for each of the IVs [41]. However, since IVW is susceptible to horizontal pleiotropy or invalid IVs, the MR-Egger method is used to complement the IVW result [42, 43]. MR-Egger is to allow all IVs to have a directed multiplicity of effects [44]. It applies InSIDE, assuming all IVs are invalid. So, it tends to cause a loss of power [41]. We also used the other three median-based measures and thought that over half of the IVs were valid to provide a precise causal estimate [44]. When the multiplicity of IVs was not present, often, the three models provided consistent estimates [45]. Later we used the Q statistic to calculate the heterogeneity and directional pleiotropy of IVs. If there were directional pleiotropy in the IVs, we would use MR-PRESSO for screening. When outliers cannot be eliminated, the causal inference role of the weighted median model should be emphasized because it has slightly lower estimation accuracy but is inherently robust to heterogeneous outliers [46]. We also use funnel plots and scatter plots for visualization. Leave-One-Out sensitivity analysis was performed to determine the effect of each SNP on the outcome. We calculated statistical efficacy using an online calculator (https://sb452.shinyapps.io/power) with a significance level of 0.05 [47]. The efficacy of the IVs related to glucocorticoid medication use was 100% and the efficacy of the IVs related to the iridocycline was 72.3%, which had a small impact on the results as the F-values of the IVs were all greater than 100, and the results of these calculations are shown in Table 3.

**Table 3:** Power (two-sided α=0.05) for conventional Mendelian randomization analysis.

**Exposure**	**Actual N (senile cataracts -GWAS)**	**Proportion of cases (senile cataracts -GWAS)**	**Observational OR**	**R^2^ of instrument**	**Power at actual N**
Glucocorticoiden	216362	0.14	1.10	0.34	1.0
Iridocyclitis	216362	0.14	1.03	0.32	0.7

R2, coefficient of determination of exposure on genetic variants; OR, odds ratio.

For MVMR analyses, we simultaneously considered the effects of iridocyclitis with prescription glucocorticoid medication use and corrected for risk factors. We performed SNP extraction for each GWAS to construct IVs, and the screening criteria were consistent with those used for univariate analyses. We removed related and duplicate SNPs (within 10 000 kilobase pairs; R2 ≥0.001). In order to eliminate correlations (multicollinearity) between exposure factors to make the outcomes more precise, we used LASSO regression analyses to screen for valid IVs and then applied the MVMR extension of the inverse variance weighted MR method. The MVMR extension of the MR-Egger method was used to correct for measured and unmeasured pleiotropy.

We identify a causal relationship between iridocyclitis, glucocorticoid drug use, and senile cataract. The results contribute to a better understanding of the role of iridocyclitis and glucocorticoid medication use in cataract progression and are informative for future selection of therapeutic agents.

## Supplementary Material

Supplementary Figures

Supplementary Tables
