# Supplementary material for: Prescription glucocorticoid medication and iridocyclitis are associated with an increased risk of senile cataract occurrence: a Mendelian randomization study
Source: Aging (Albany NY). 2024 Jun 26;16(12):10563–78. doi: 10.18632/aging.205963 (PMC11236313; doi:10.18632/aging.205963)
Supplement: Supplementary Figures [file aging-16-205963-s001.pdf]

SUPPLEMENTARY FIGURES

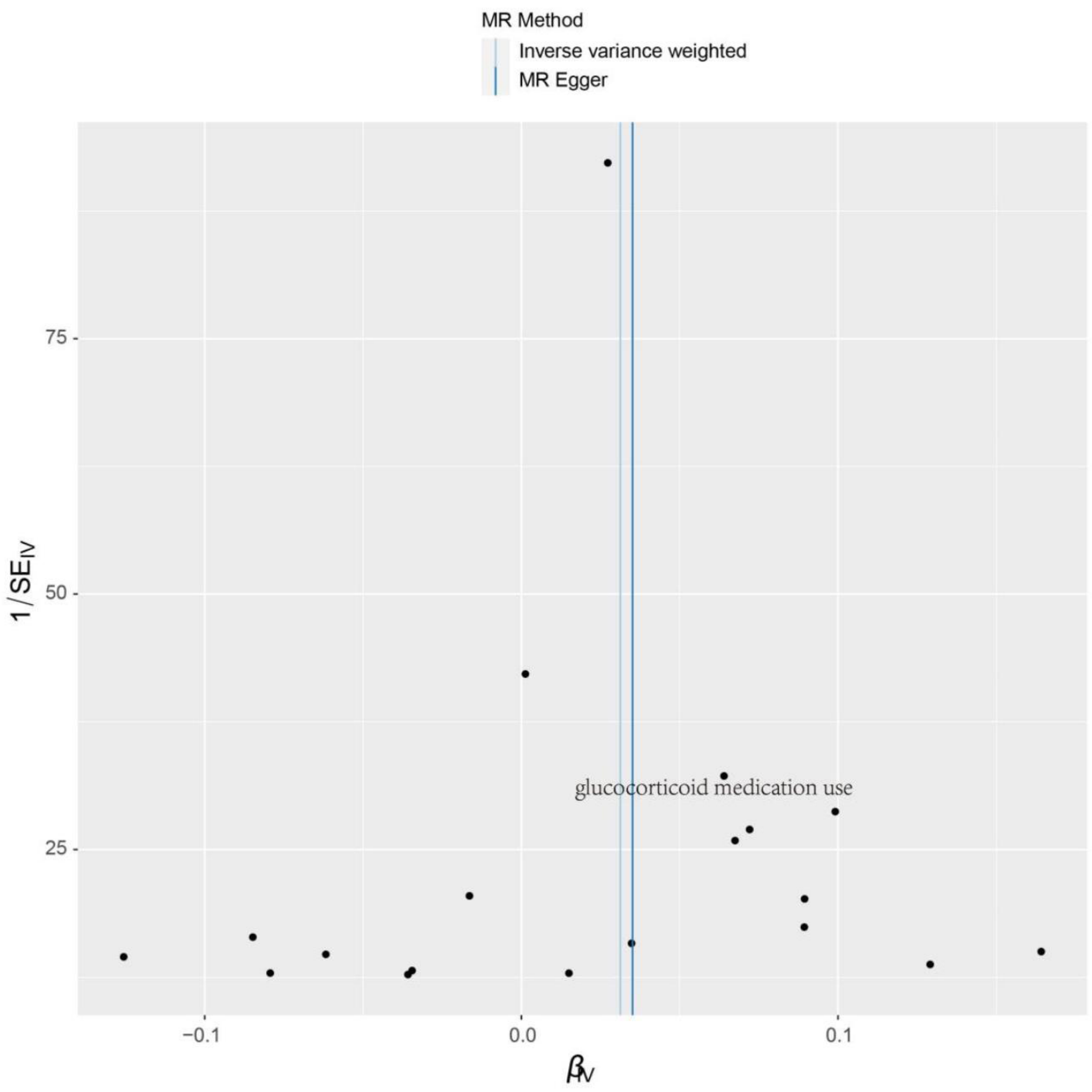

Supplementary Figure 1. Scatter plot of the effect of iridocyclitis on cataracts. Each black point represents a SNP, plotted by the estimate of SNP on iridocyclitis and the estimate of SNP on the risk of cataracts. MR, Mendelian randomization; SNP, single nucleotide polymorphism.

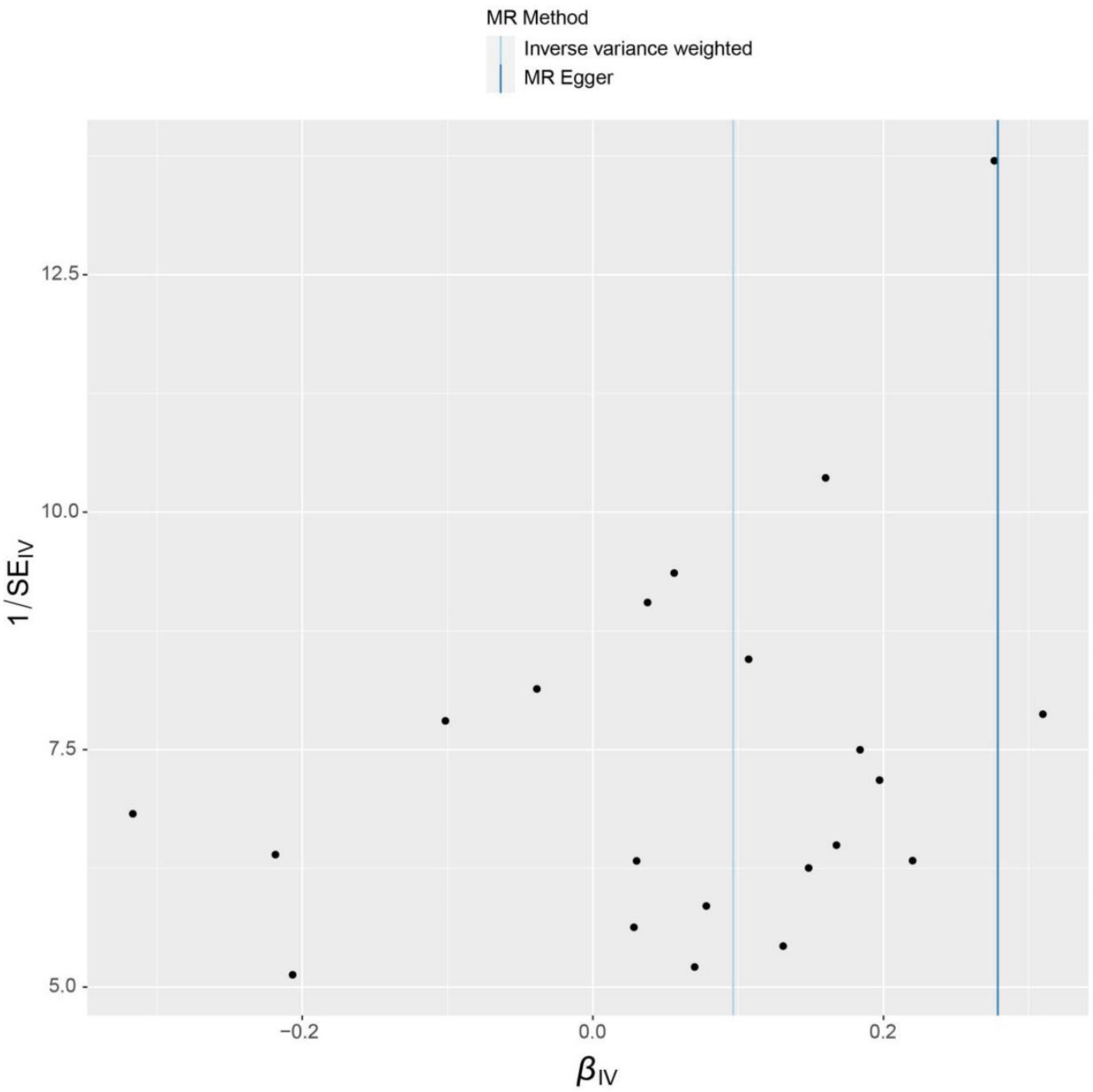

Supplementary Figure 2. Scatter plot of the effect of iridocyclitis on cataracts. Each black point represents a SNP, plotted by the estimate of SNP on glucocorticoid medication use and the estimate of SNP on the risk of cataracts. MR, Mendelian randomization; SNP, single-nucleotide polymorphism.

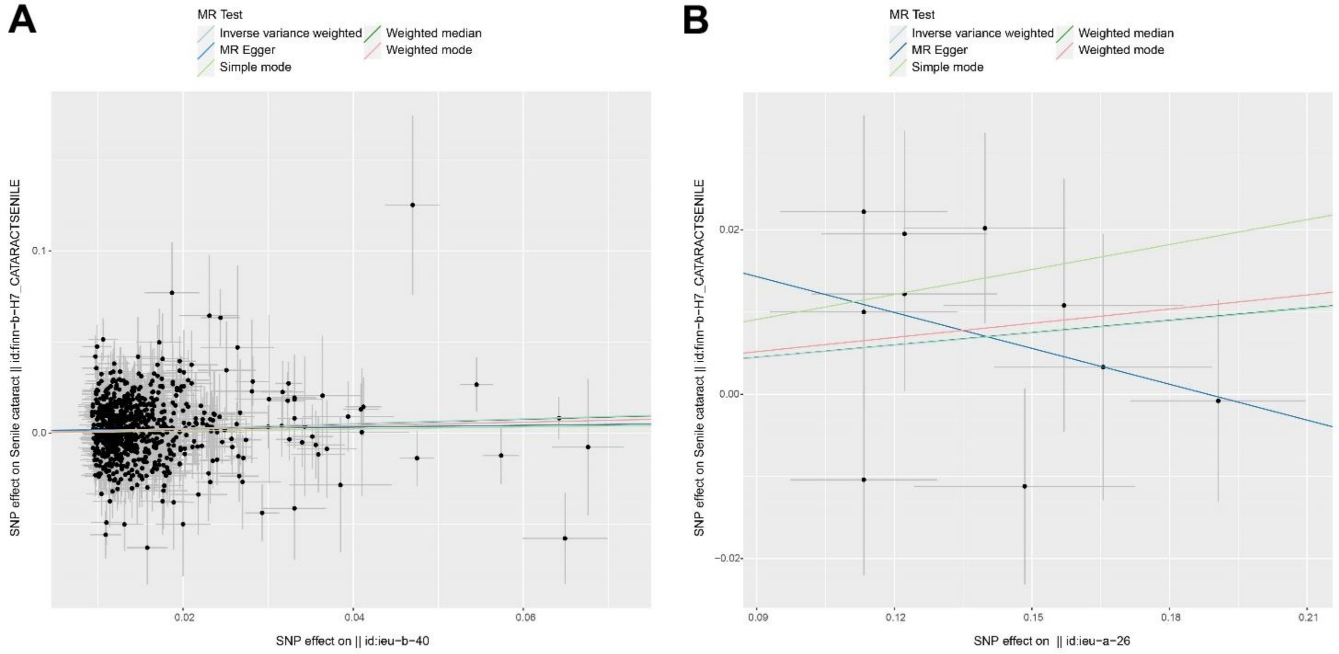

Supplementary Figure 3. Scatter plots for MR analyses of BMI and T2D on BMDs. Scatter plots for MR analyses of the causal effect of BMI (**A**) and T2D (**B**) on BMDs. The slope of the straight-line fitting of the scatterplot shows the magnitude of the causal relationship.
