# Supplementary material for: Prescription glucocorticoid medication and iridocyclitis are associated with an increased risk of senile cataract occurrence: a Mendelian randomization study
Source: Aging (Albany NY). 2024 Jun 26;16(12):10563–78. doi: 10.18632/aging.205963 (PMC11236313; doi:10.18632/aging.205963)
Supplement: Supplementary Tables [file aging-16-205963-s002.pdf]

SUPPLEMENTARY TABLES

Supplementary Table 1. Instrumental variables indicating iridocyclitis.

SNP	Effect allele exposure	Other allele exposure	Beta exposure	Eaf exposure	Chr	Pos	Se exposure	Pval exposure	Pos exposure	Sample size exposure
rs114969413	A	G	0.4953	0.0314	6	31925679	0.0731	1.21E- 11	31925679	212909
rs12660597	G	A	0.3172	0.1678	6	33847531	0.0336	3.60E-21	33847531	212909
rs16891359	C	T	0.2063	0.1763	6	26149000	0.0337	9.51E- 10	26149000	212909
rs17206617	G	A	1.9456	0.08176	6	31378640	0.0521	1.00E-200	31378640	212909
rs181316459	C	G	0.4647	0.04744	7	5473610	0.0614	3.84E- 14	5473610	212909
rs2549803	T	C	-0.1798	0.3345	5	96174929	0.0261	5.35E- 12	96174929	212909
rs3104418	A	G	0.3316	0.373	6	32581021	0.0262	8.09E-37	32581021	212909
rs34119722	C	G	0.3548	0.05313	6	24095141	0.0553	1.42E- 10	24095141	212909
rs3871467	A	G	0.3053	0.4428	6	29677249	0.025	3.27E-34	29677249	212909
rs3993757	T	C	1.0594	0.02452	6	31590746	0.0869	3.53E-34	31590746	212909
rs62392759	A	C	0.2194	0.1349	6	25804685	0.036	1.10E-09	25804685	212909
rs62443225	A	G	0.282	0.07947	7	5482137	0.047	1.95E-09	5482137	212909
rs74853132	T	C	0.5203	0.03314	6	35537065	0.0714	3.06E- 13	35537065	212909
rs7772289	T	G	-0.246	0.6169	6	28674322	0.0258	1.75E-21	28674322	212909
rs77831243	C	T	0.5103	0.02366	6	25170013	0.0846	1.62E-09	25170013	212909
rs879882	C	T	-0.1768	0.6175	6	31139452	0.0259	8.05E- 12	31139452	212909
rs9277557	C	T	0.2541	0.1959	6	33056694	0.0313	4.92E- 16	33056694	212909
rs9295932	G	A	0.4282	0.2564	6	30870463	0.029	3.17E-49	30870463	212909
rs9468364	A	T	0.9406	0.07333	6	28355711	0.0508	1.52E-76	28355711	212909

SNP	Beta outcome	Eaf outcome	Se outcome	Samplesize outcome	Pval. Outcome	r2	F
rs114969413	0.0813	0.03145	0.033	216362	0.01359	0.002894308	628.0301746
rs12660597	-0.0052	0.168	0.0155	216362	0.7358	0.012110881	2652.433538
rs16891359	0.0031	0.1763	0.016	216362	0.8486	0.005113301	1111.999876
rs17206617	0.0531	0.08178	0.0211	216362	0.0119501	0.110092521	26766.39796
rs181316459	-0.0394	0.04743	0.0283	216362	0.1628	0.004293294	932.9024143
rs2549803	0.0111	0.3345	0.0122	216362	0.3652	0.008333787	1818.251013
rs3104418	0.0239	0.3728	0.0123	216362	0.0513902	0.0274816	6113.939759
rs34119722	0.0458	0.05314	0.0258	216362	0.0764892	0.003428619	744.3682041
rs3871467	0.0206	0.443	0.0118	216362	0.0809599	0.026831241	5965.262618
rs3993757	0.105	0.02455	0.0369	216362	0.00438904	0.007842602	1710.238163
rs62392759	-0.0174	0.1349	0.017	216362	0.3043	0.004745902	1031.719715
rs62443225	-0.0101	0.07943	0.0221	216362	0.6484	0.003527678	765.9503665
rs74853132	0.0181	0.03305	0.0329	216362	0.582	0.00342564	743.7192145
rs7772289	-0.022	0.6169	0.0122	216362	0.0703704	0.016025586	3523.76617
rs77831243	-0.0176	0.02363	0.0388	216362	0.651	0.001983794	430.0669144
rs879882	0.0222	0.6175	0.0122	216362	0.0695697	0.008246845	1799.124487
rs9277557	0.0227	0.1959	0.0146	216362	0.1202	0.009638052	2105.582696
rs9295932	0.0274	0.2564	0.0133	216362	0.0390796	0.033580266	7517.878761
rs9468364	0.0012	0.07321	0.0223	216362	0.9577	0.030248125	6748.617246

beta, MR effect estimate; eaf, effect allele frequency; chr, chromosome; pos, base pair position, se, standard error of effect estimate, pval, p-value of effect estimate; F, F statistics (assessing the extent of weak instrumental variable bias), r2, r2 statistics (assessing the level of IVs interpretation exposure).

Supplementary Table 2. Instrumental variables indicating glucocorticoid medication use.

SNP	Effect allele exposure	Other allele exposure	Beta exposure	Eaf exposure	Chr	Pos	Se exposure	Pval exposure	Samplesize exposure
rs1011082	C	T	0.07408	0.483207	17	38068514	0.011180029	3.40E- 11	205700
rs10154834	C	T	-0.067905188	0.494958	3	33068055	0.011217121	1.40E-09	205700
rs117710327	A	C	-0.141427443	0.0620109	19	33726578	0.023380492	1.50E-09	205700
rs12123821	T	C	0.153392807	0.0470148	1	152179152	0.026356427	5.90E-09	205700
rs1391371	T	A	0.204121963	0.202145	6	32603798	0.013949579	1.70E-48	205700
rs1504215	A	G	-0.086968465	0.350757	6	91006227	0.011709394	1.10E- 13	205700
rs1689510	C	G	0.080533166	0.338025	12	56396768	0.011856495	1.10E- 11	205700
rs1775553	T	C	-0.117927194	0.423019	10	9054325	0.011302505	1.70E-25	205700
rs1898671	T	C	0.097538431	0.350684	5	110408002	0.011702287	7.80E- 17	205700
rs1963497	A	C	-0.097599281	0.182496	15	61071791	0.014568562	2.10E- 11	205700
rs2287037	T	C	0.096040754	0.395554	2	102979028	0.011432775	4.40E- 17	205700
rs2517544	G	C	0.093034136	0.321999	6	31009508	0.011951148	7.00E- 15	205700
rs2949661	T	C	-0.074315371	0.401994	1	167424924	0.011428852	7.90E- 11	205700
rs340931	G	A	-0.06567395	0.324864	9	6086443	0.011922614	3.60E-08	205700
rs34290285	A	G	-0.12759555	0.255506	2	242698640	0.012807354	2.20E-23	205700
rs35441874	A	T	-0.077133744	0.246038	16	11213021	0.013065188	3.60E-09	205700
rs4739738	G	A	0.078805425	0.358014	8	81291645	0.011672134	1.50E- 11	205700
rs72743461	A	C	0.09897992	0.236679	15	67441750	0.013141574	5.00E- 14	205700
rs7936312	T	G	0.098884502	0.477022	11	76293726	0.011205948	1.10E- 18	205700
rs9270902	A	G	-0.114948411	0.274952	6	32571962	0.012518021	4.20E-20	205700
rs992969	A	G	0.139871247	0.251777	9	6209697	0.012898849	2.10E-27	205700

SNP	Beta outcome	Eaf outcome	Se outcome	Samplesize outcome	Pval outcome	r2	F
rs1011082	0.0163	0.4478	0.0117	216362	0.1618	0.01212344	3927.086278
rs10154834	-0.0053	0.5154	0.0116	216362	0.6463	0.010106486	3267.074091
rs117710327	-0.0279	0.09883	0.0197	216362	0.1563	0.004866735	1564.961749
rs12123821	-0.0317	0.03956	0.0299	216362	0.2896	0.003999998	1285.131801
rs1391371	0.0564	0.1937	0.0149	216362	0.000151799	0.045771845	15349.47253
rs1504215	0.019	0.2397	0.0136	216362	0.1615	0.014508524	4711.049168
rs1689510	0.0135	0.3185	0.0124	216362	0.2777	0.01201383	3891.149024
rs1775553	-0.0066	0.3025	0.0126	216362	0.6011	0.029219637	9631.658858
rs1898671	-0.0099	0.3051	0.0125	216362	0.4302	0.018214034	5936.583506
rs1963497	0.0309	0.2041	0.0143	216362	0.0309799	0.009535253	3080.636542
rs2287037	-0.0037	0.4003	0.0118	216362	0.756201	0.018923824	6172.391122
rs2517544	0.0028	0.2226	0.0147	216362	0.8486	0.015598332	5070.526645
rs2949661	-0.0021	0.26	0.0132	216362	0.8709	0.011428977	3699.531758
rs340931	-0.0046	0.3005	0.0126	216362	0.7177	0.007890084	2544.890559
rs34290285	-0.0048	0.2165	0.0141	216362	0.7364	0.023645599	7749.792972
rs35441874	-0.0101	0.2123	0.0142	216362	0.4775	0.00826773	2667.713091
rs4739738	0.0117	0.3073	0.0126	216362	0.3517	0.012057112	3905.338856
rs72743461	0.0182	0.2624	0.0132	216362	0.167	0.013310143	4316.674759
rs7936312	0.0106	0.4156	0.0117	216362	0.3666	0.021253521	6948.770024
rs9270902	-0.0356	0.3337	0.0146	216362	0.0148399	0.020615886	6735.908864
rs992969	0.0224	0.2377	0.0135	216362	0.0987302	0.027698422	9115.936746

beta, MR effect estimate; eaf, effect allele frequency; chr, chromosome; pos, base pair position; se, standard error of effect estimate; pval, p-value of effect estimate; F, F statistics (assessing the extent of weak instrumental variable bias). r2, r2 statistics (assessing the level of IVs interpretation exposure).
